# Supplementary material for: Unsupervised Clustering of Heartbeat Dynamics Allows for Real Time and Personalized Improvement in Cardiovascular Fitness
Source: Sensors (Basel). 2022 May 24;22(11):3974. doi: 10.3390/s22113974 (PMC9182749; doi:10.3390/s22113974)
Supplement: Supplementary file 1 [file sensors-22-03974-s001.zip › sensors-1703216-supplementary.pdf]

Supplementary Materials Figure S1

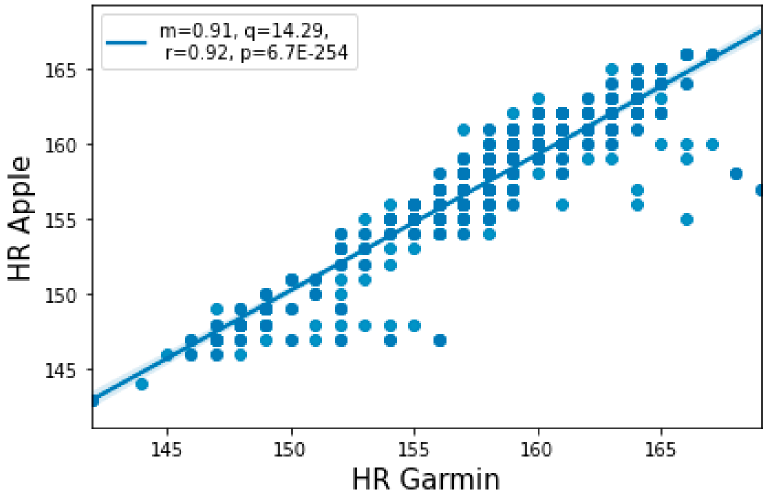

Figure S1. Comparison analysis between Heart Rate acquired by Apple Watch and Heart Rate acquired by Garmin. Linear regression highlights a Pearson correlation coefficient R = 0.92 and p value << 0.0001 so we can say that the two signals are highly correlated.

Supplementary Materials Figure S2

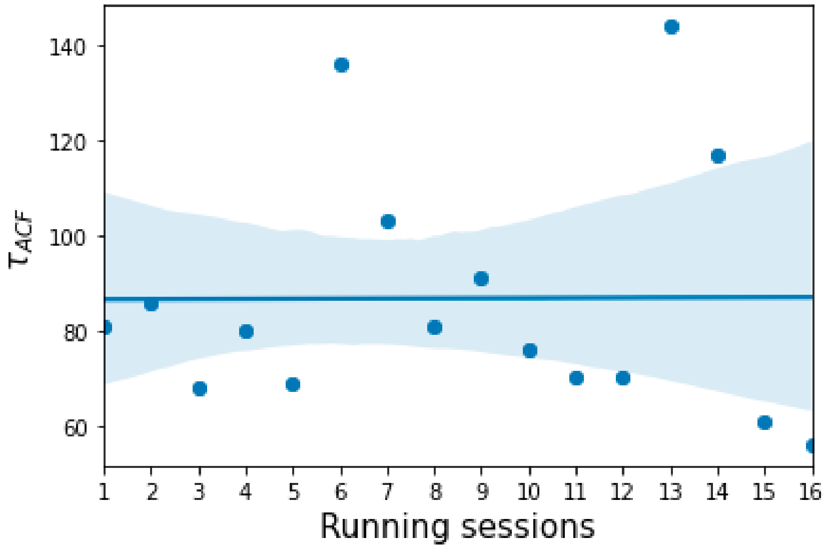

Figure S2. ACF time decay (τACF) values in sixteen different running sessions acquired by Garmin. τACF values have been obtained by calculating the intersection values of the ACF with confidence intervals. An important result is the independence of the ACF time decay from training level. The plot in Figure S2 does not show any trend meaning that τACF is not correlated with physical fitness.

Supplementary Materials Figure S3

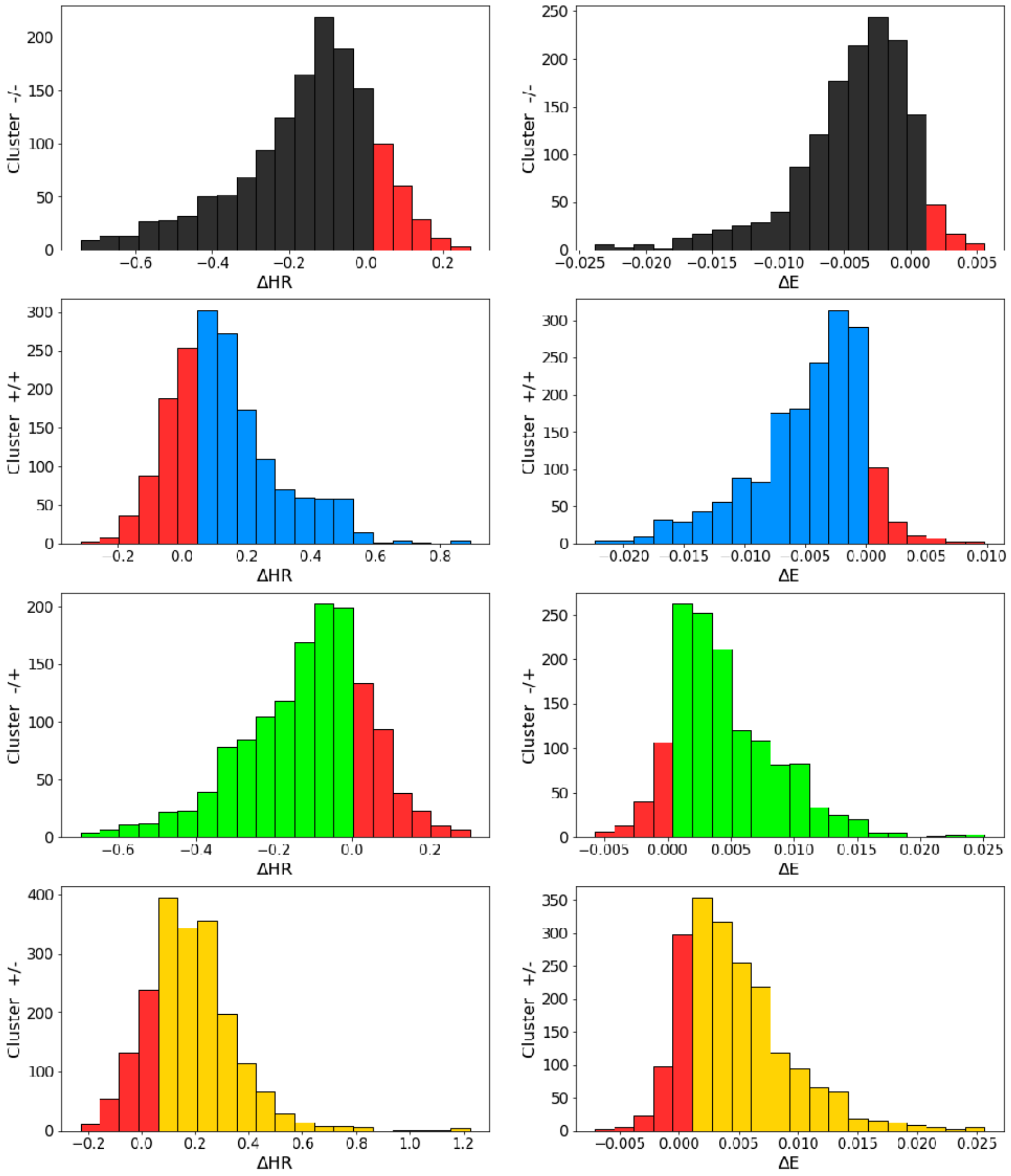

Figure S3. Slopes Distributions obtained from the linear regression analysis relative to the four clusters and to the two clustering features ΔE and ΔHR are reported. The red bars indicate the slope values whose sign do not correspond to the sign of the variations of the clustering analysis, ranging 8% and 23% of the points. The colors of the bar plots are equal to the color used to indicate the respective clusters (black for -/- cluster, blue for -/+ cluster, green for +/- cluster and yellow for +/+ cluster (see Figure 2 in Section 3.1.)

Supplementary Materials Figure S4

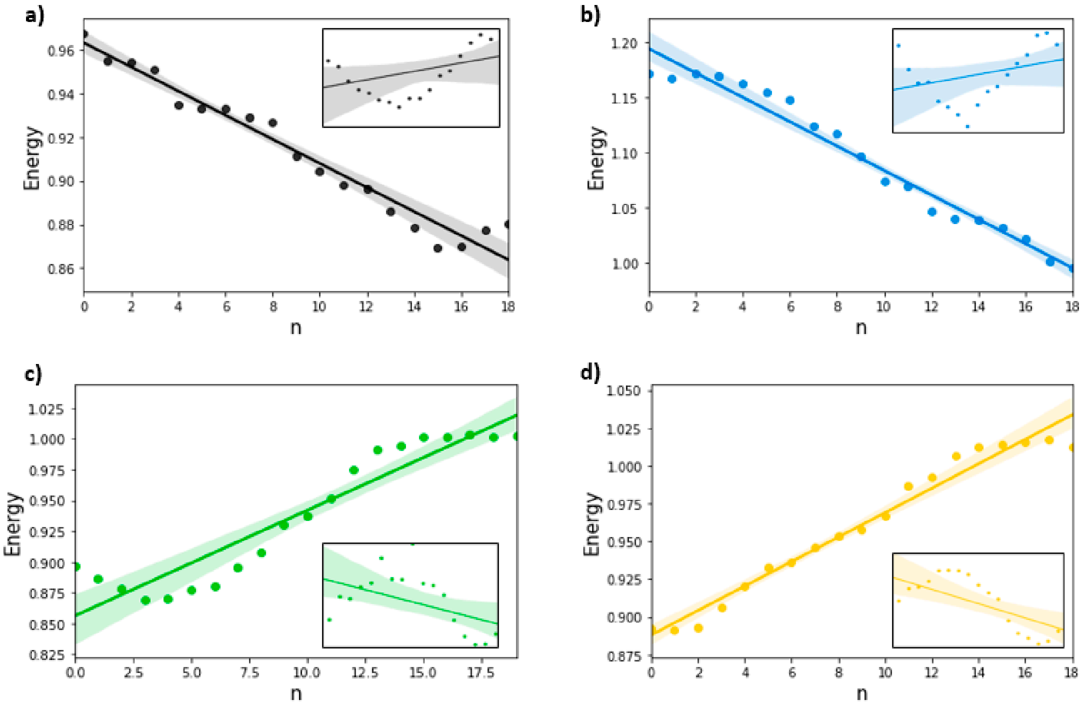

Figure S4. Energy plots representing cases in which the sign of the variations of the features chosen in our clustering analysis (ΔE and ΔHR, according to equation 7) and the sign of the slope calculated with the linear analysis are in agreement. The colors of the plots are equal to the color used to indicate the respective clusters (black for -/- cluster, blue for -/+ cluster, green for +/- cluster and yellow for +/+ cluster (see Figure 2 in Section 3.1.). In the inserts are instead reported energy plots representing representative cases in which the value of the variations of the features chosen in our clustering analysis and the value of the slope calculated with the linear analysis disagree. For example, in the inset plots in Figure S4a,b the energy variation (ΔE) should be negative. Linear regression, on the other hand, identifies a positive slope. Similarly in Figure S4c,d ΔE should be negative but the slopes are positive. This happens when there are particular configurations in which the variation is very close to zero. In these cases the slope becomes sensitive to noise. Instead in our features the value of γ_0 is close to zero and therefore the skewness γ_1 becomes important. Therefore in these cases the sign of the variation takes into account the general tendency of the data to be above or below the average value.

Supplementary Materials Table S1

Table S1. Values of repeated measures ANOVA and Tukey HSD post hoc for both RQA and Clustering analysis performed on the three temporal sections. *p*-value annotation legend: ns: $5.00 \times 10^{-2} < p <= 1.00 \times 10^{0}$; *: $1.00 \times 10^{-2} < p <= 5.00 \times 10^{-2}$; **: $1.00 \times 10^{-3} < p <= 1.00 \times 10^{-2}$; ***: $1.00 \times 10^{-4} < p <= 1.00 \times 10^{-3}$; ****: $p <= 1.00 \times 10^{-4}$.

Analysis	Features	section1 N=21	section2 N=21	section3 N=21	P-value	Post-hoc pairwise comparison (padj)		
		Mean±sd	Mean±sd	Mean±sd	p	Start-middle	Start-end	Middle-end
RQA	DET	0.86±0.06	0.90±0.05	0.92±0.02	p<0.0001 (****)	0.0234 (*)	p<0.001 (***)	0.2791
Clustering	%- -	0.28±0.07	0.22±0.06	0.24±0.07	0.0198 (*)	0.0143 (*)	0.0996	0.6869
	%-+	0.33±0.06	0.27±0.06	0.22±0.07	p<0.0001 (****)	0.0079 (**)	p<0.001 (***)	0.0447 (*)
	%+-	0.15±0.06	0.22±0.08	0.21±0.07	0.0032 (**)	0.0032 (**)	0.011 (*)	0.9
	%++	0.23±0.07	0.28±0.07	0.33±0.06	p<0.001 (***)	0.038 (*)	p<0.001 (**)	0.1163
